# Augmentation of histone deacetylase 3 (*HDAC3*) epigenetic signature at the interface of proinflammation and insulin resistance in patients with type 2 diabetes

**DOI:** 10.1186/s13148-016-0293-3

**Published:** 2016-11-24

**Authors:** Chandrakumar Sathishkumar, Paramasivam Prabu, Mahalingam Balakumar, Raji Lenin, Durai Prabhu, Ranjith Mohan Anjana, Viswanathan Mohan, Muthuswamy Balasubramanyam

**Affiliations:** Department of Cell and Molecular Biology and Dr. Rema Mohan High-Throughput Screening (HTS) Lab, Madras Diabetes Research Foundation and Dr. Mohan’s Diabetes Specialties Centre, Gopalapuram, Chennai, 600086 India

**Keywords:** HDAC3, Inflammation, Insulin resistance, Type 2 diabetes, Epigenetics, Sirt1, Histone modification

## Abstract

**Background:**

A role of proinflammation has been implicated in the pathogenesis of diabetes, but the up-stream regulatory signals and molecular signatures are poorly understood. While histone modifications such as changes in histone deacetylase (HDAC) are emerging as novel epigenetic biomarkers, there is lack of studies to demonstrate their clinical relevance in diabetes. Therefore, we investigated the extent of HDAC machinery and inflammatory signals in peripheral blood mononuclear cells (PBMCs) from patients with type 2 diabetes mellitus (T2DM) compared to control subjects.

**Results:**

*HDAC3* activity was significantly (*p* < 0.05) increased in patients with T2DM compared to control subjects. While subtypes of HDACs were differentially expressed at their transcriptional levels in patients with type 2 diabetes, the most prominent observation is the significantly (*p* < 0.05) elevated messenger RNA (mRNA) levels of *HDAC3*. Expression levels of Sirt1 which represents the class III HDAC were decreased significantly in T2DM (*p* < 0.05). Plasma levels of both TNF-α and IL-6 were significantly higher (*p* < 0.05) in patients with type 2 diabetes compared to control subjects. Among the proinflammatory mediators, the mRNA expression of MCP-1, IL1-β, NFκB, TLR2, and TLR4 were also significantly (*p* < 0.05) increased in T2DM. Transcriptional levels of DBC1 (deleted in breast cancer 1, which is a negative regulator of HDAC3) were seen significantly reduced in PBMCs from T2DM. Interestingly, *HDAC3* activity/HDAC3 mRNA levels positively correlated to proinflammation, poor glycemic control, and insulin resistance.

**Conclusions:**

Striking message from this study is that while looking for anti-inflammatory strategies and drugs with novel mode of action for T2DM, discovering and designing specific inhibitors targeted to *HDAC3* appears promising.

## Background

More than 415 million people are estimated to have diabetes worldwide now, and the prevalence of diabetes in India alone is estimated to be 69.2 million, with ever increasing morbidity and mortality [1]. Asian Indians have unique clinical and biochemical abnormalities which makes them more prone to diabetes and premature coronary artery diseases [2, 3]. Low-grade chronic inflammation and insulin resistance appear to play a key role in the pathogenesis of diabetes and its major vascular complications [4, 5]. Serum levels of inflammatory and chemokine markers have not only been strongly associated but also appear to be independent predictors of type 2 diabetes [4–6]. We have demonstrated increased proinflammatory markers and ER stress accompanied by impaired miR-146a in patients with type 2 diabetes that showed association with several metabolic risk factors [7, 8]. Although proinflammation has been linked to insulin resistance and development of type 2 diabetes, the underlying and up-stream molecular and cellular mechanisms remain enigmatic.

Interactions of environmental factors with genetic and epigenetic variants are likely to play an important role in the pathogenesis of type 2 diabetes [9]. Aberrant epigenetic modifications such as DNA methylation, histone modification, and microRNA alterations are well recognized drivers for the cancer phenotype, but the accumulating evidence also imply their role in the etiology of diabetes and cardiovascular diseases. In recent years, histone deacetylases (HDACs) have received increasing attention in the context of several disease states including type 2 diabetes [10] and they are considered as emerging druggable targets. Based upon their yeast sequence homology, HDACs are classified into four groups namely, class I (HDAC1, 2, 3, and 8), class II (HDAC4, 5, 6, 7, 9, and 10), class III (sirtuins 1–7), and class IV (HDAC11). As HDAC modulates the acetylation status of histone and other important nonhistone cellular proteins, they have been recognized as potential therapeutic targets for a broad range of human disorders [11] and inhibition of HDAC has been suggested as a new therapeutic intervention of potential importance in type 2 diabetes [12].

Although clinical evidence on the impact of different classes of HDACs on metabolic risks are only emerging, works on animal and cell models suggest HDAC inhibition as a potential therapeutic avenue. With special reference to the skeletal muscle and adipose tissue, class I selective HDAC inhibition has been shown to increase expression of several key mitochondria-related transcription factors, as well as the levels of multiple genes involved in glucose and lipid metabolism [13]. Class I HDAC inhibitor MS-275 prevents β-cell death elicited by cytokines and prevented from apoptosis induced by saturated free fatty acid palmitate treatment associated with reduced endoplasmic stress marker CHOP and ATF3 [14]. On the other hand, sirtuin1 (Sirt1, a class III HDAC) which is a well-known metabolic regulator of glucose homeostasis and insulin resistance-associated inflammation is reduced in diabetes, and enhanced activity of sirt1 has been shown to be protective against diabetes [15]. Since different HDACs have distinct role in various metabolic pathways, signaling, and functions, the idea of pan HDAC inhibition may not work effectively with minimal/zero side effects. Therefore, elucidating the individual HDAC status, function, and its clinical relevance to metabolic pathways is the need of the day. While Asian Indians are more prone to develop type 2 diabetes and other vascular complications, there is lack of data on epigenetic signatures in the context of clinical diabetes setting. Therefore, in this study, we used peripheral blood mononuclear cells (PBMCs) as a surrogate cell model to delineate the activity and transcriptional levels of different HDACs and to correlate their association with glycemia, proinflammation, and insulin resistance in patients with type 2 diabetes.

## Methods

### Study design and methods

Study subjects (both males and females) were recruited from the on-going epidemiological studies at the Madras Diabetes Research Foundation and Dr. Mohan’s Diabetes Specialties Centre, Chennai, India. With a pilot study and considering the primary outcome variables (HDAC3 activity), the required sample size for the study was calculated to be *n* = 22 in each arm in order to have 80% statistical power with an alpha error of 5%. Therefore, the study comprised of two groups (*n* = 25 each) viz., subjects with normal glucose tolerance (NGT) and patients with type 2 diabetes mellitus (T2DM). Subjects were characterized either as NGT or T2DM according to WHO criteria. Of the diabetic patients, 92% of them were on oral hypoglycemic agents (OHA) and 8% were on insulin in addition to OHA. The study has been carried out in accordance with the guidelines of Declaration of Helsinki and was approved by the Institutional Ethics Committee of the Madras Diabetes Research Foundation, and informed written consent has been obtained from all the study subjects.

### Anthropometric measurements

Anthropometric measurements including height, weight, and waist circumstance were obtained using standardized techniques. Height was noted with a tape measured to the nearest centimeter. Weight was measured with traditional spring balance that was kept on a firm horizontal surface. The body mass index (BMI) was calculated using the formula, weight (kg)/height (m)^2^. Waist circumference was measured using a non-stretchable fiber measuring tape. Blood pressure was recorded from the right arm of study subjects when they were relaxed and in sitting position to the nearest 2 mmHg with a mercury sphygmomanometer (Diamond Deluxe BP apparatus, Pune, India). Two reading were taken 5 min apart, and the mean of the two was taken as the blood pressure.

### Biochemical investigations

Fasting plasma glucose (glucose oxidase-peroxidase method), serum cholesterol (cholesterol oxidase-peroxidase-amidopyrine method), serum triglycerides (glycerol phosphate oxidase-peroxidase-amidopyrine method) and HDL cholesterol (direct method-polyethylene glycol-pretreated enzymes) were measured using Hitachi-912 Autoanalyser (Hitachi, Mannheim, Germany). The intra- and inter-assay co-efficient of variation for the biochemical assays were <5%. Low-density lipoprotein (LDL) cholesterol was calculated using the Friedewald formula [16]. Glycated hemoglobin (HbAlc) was estimated by high-pressure liquid chromatography using the variant analyzer (Bio-Rad, Hercules, Calif., USA). Serum insulin was estimated using enzyme-linked immunosorbent assay (Calbiotech, CA). The intra-assay and the inter-assay coefficients of variation for insulin assay was <10%. Insulin resistance was calculated using the homeostasis assessment model (HOMA-IR) using the formula: fasting insulin (μIU/mL)*fasting glucose (mmol/L) / 22.5.

### Blood collection and PBMC preparation

About 8 to 10 mL of fasting (8–12 h), venous blood was collected in the anticoagulant (ACD) vacutainer from the study group subjects according to the standard procedures. All fresh blood collected was processed immediately within 2 h from the time of collection. Plasma was separated from the whole blood by centrifuging tubes at 3000 rpm for 20 min at room temperature and stored at −80 °C until analyzed for cytokines. Blood was also processed for PBMC isolation using Histopaque-1077 (Sigma-Aldrich) according to the standard protocol by overlaying the blood on density gradient solution and centrifuged at 1500–1800 rpm for 30 min. The Buffy coat layer containing the peripheral blood mononuclear cells (PBMCs) was collected, washed thrice with phosphate-buffered saline (PBS, pH 7.2–7.4), and used immediately and separately for nuclear protein extraction and total RNA isolation.

### Fractionation of nuclear extract

Nuclear protein was extracted from freshly isolated PBMCs using ProteoJET Cytoplasmic and Nulcear Protein Extraction Kit (Fermentas, Canada). Briefly, 8 × 10^6^ PBMCs were suspended in 300 μL of cell lysis buffer, vortexed for 10 s, and incubated for 10 min at 4 °C. After the first round of centrifugation at 500×*g* for 7 min, the supernatants were separated as cytoplasmic fraction and the pellet was processed as nuclear extract. The pellet was rinsed twice with nuclei washing buffer, resuspended in nuclei storage buffer, and subsequently treated with nuclei lysis reagent. After an incubation for 20 min on ice with repetitive vortexing done every 3 min, the resulting nuclear extract were centrifuged at 20,000×*g* for 5 min at 4 °C; supernatants were collected as nuclear fractions and frozen at −80 °C for later analysis.

### Global HDAC/specific HDAC3 activity assay

Global HDAC activity in PBMC nuclear extract was assessed using a Fluorometric HDAC Activity Assay Kit (Biovision, CA). All PBMC samples were incubated with HDAC substrate (Boc-Lys(Ac)-AMC). Deacetylation of substrate sensitizes substrate, and further addition of lysine developer produces a fluorophore, which was captured as the fluorescence read-out using a plate reader with Ex. = 350–380 nm and Em. = 440–460 nm settings. The standard curve was prepared as per recommended dilution range by the kit protocol. The absolute HDAC activity was calculated based on the slope determined by the standard deacetylated curve and expressed as μM/mL. Positive and negative controls were carried out during every experiment. Specific *HDAC3* activity was assessed using the *HDAC3* activity assay kit (Biovision, CA) as per the manufactures’ protocol. Absolute *HDAC3* activity was calculated based on the slope determined by the standard deacetylated curve and expressed as pmol/min/mL.

### Total RNA isolation and cDNA preparation

Transcriptional levels of specific gene alterations in PBMCs were studied in a subset (*n* = 14 each) of the NGT and T2DM groups. Total RNA was isolated using TRIzol reagent (Invitrogen). RNA quantity and quality were assessed by NanoDrop 2000 (Thermo scientific) instrument. Then, 1 μg of total RNA was taken for the first-strand complementary DNA (cDNA) synthesis reaction. Briefly, RNA was adjusted with nuclease-free water and mixed with cDNA synthesis master mix (NEB) containing 100 units of M-MuLV reverse transcriptase enzyme and 2X buffer, 40 μM oligo dT and random hexamer primer mix, 20 units of RNase inhibitor, and 10 mM dNTPs solution mix. The resultant samples were incubated at 42 °C for 60 min for first-strand cDNA synthesis followed by 5 min at 95 °C for enzyme deactivation. cDNA reaction negative control without reverse transcriptase enzyme (−RT) was performed with the experiment.

### Quantitative real time PCR

The relative expression of the messenger RNA (mRNA) was analyzed by preparing reaction mixer with Power SYBR Green PCR Master Mix (2X) (Applied Biosystems) and gene specific primers with diluted cDNA and final volume made up to 20 μL with nuclease-free water. Quantification and analysis were carried out in ABI prism 7000 (Applied Biosystems) real time PCR. The target gene expression was normalized to the house keeping gene β-actin, and relative expression was determined using Delta Delta Ct (DDCt) method. Nontemplate control (NTC) was performed for each reaction assay plates.

### Plasma TNF-α and IL-6 measurements (ELISA)

Plasma tumor necrosis factor-α (TNF-α) and interleukin-6 (IL-6) levels were measured by quantitative sandwich enzyme-linked immunosorbent assay (ELISA) (eBioscience, CA, USA). In brief, capture antibody specific for TNF-α or IL-6 was coated in the microplate a day prior to the experiment, sealed, and incubated overnight at 4 °C. The next day, plate was washed with washing buffer and standards or samples added to the appropriate wells. After incubation at room temperature for 2 h, TNF-α or IL-6 bound to the capture antibody and unbound was washed in the next step. Then, detection antibody followed by Avidin-HRP was added, incubated, and washed away. Finally, the color was developed after the addition of substrate solution. The reaction was stopped by stop solution and read at 450 and 570 nm. The values were expressed as pg/mL units. The intra- and inter-assay coefficients of variation were <10%.

### Statistical analysis

All data are represented as mean ± standard error mean (SEM) until otherwise mentioned as standard deviation (SD). Comparison between groups were performed using unpaired student *t* test and one-way ANOVA with *p* < 0.05 as the criterion for significance. Pearson correlation was done between variables and the risk factors. All analysis was done using IBM SPSS statistics package 20 and Graphpad prism (version 6).

## Results

### Clinical and biochemical characterization

Table 1 illustrates clinical and biochemical parameters of the study subjects. There were no significant differences in age, BMI, and waist circumferences among study groups. Systolic blood pressure was significantly higher in the T2DM patients compared to control subjects. Fasting plasma glucose (*p* < 0.001), HbAlc (*p* < 0.001), and HOMA-IR (*p* < 0.001) were significantly increased in the T2DM patients. Lipid profiles did not differ among the study subjects. This clinical characterization of our study subjects signifies that to major extent, both hyperglycemia and insulin resistance were the main clinical phenotype in type 2 diabetes compared to control subjects.

**Table 1:** Clinical and biochemical characteristics of the study subjects

Parameter	Normal glucose tolerance (NGT) (*n* = 25)	Type 2 diabetes mellitus (T2DM) patients (*n* = 25)	*p* value*
Age (years)	45.2 ± 9	45.0 ± 9	0.928
Gender–male (female)	14 (11)	16 (9)	–
Body mass index (kg/m^2^)	25.5 ± 3.5	26.6 ± 4.3	0.318
Waist circumference (cm)	94 ± 7	93 ± 11	0.964
Fasting plasma glucose (mg/dl)	94 ± 11	189 ± 46	<0.001*
Glycated hemoglobin—HbA1c (%)	5.9 ± 0.4	9.2 ± 1.7	<0.001*
HOMA-IR	2.09 ± 1	4.4 ± 1.6	<0.001*
Systolic blood pressure (mmHg)	123 ± 10	135 ± 21	<0.05*
Diastolic blood pressure (mmHg)	79 ± 8	81 ± 11	0.426
Total cholesterol (mg/dl)	171 ± 32	170 ± 48	0.953
Serum triglycerides (mg/dl)	122 ± 52	140 ± 69	0.285
HDL cholesterol (mg/dl)	41 ± 9	40 ± 8	0.389
LDL cholesterol (mg/dl)	108 ± 31	103 ± 37	0.624

Data represented as mean ± standard deviation (SD). Italic values are to indicate the statistical significance

*p value for comparison between NGT and T2DM individuals

### Altered HDAC epigenetic signatures in patients with T2DM

**Fig. 1:**
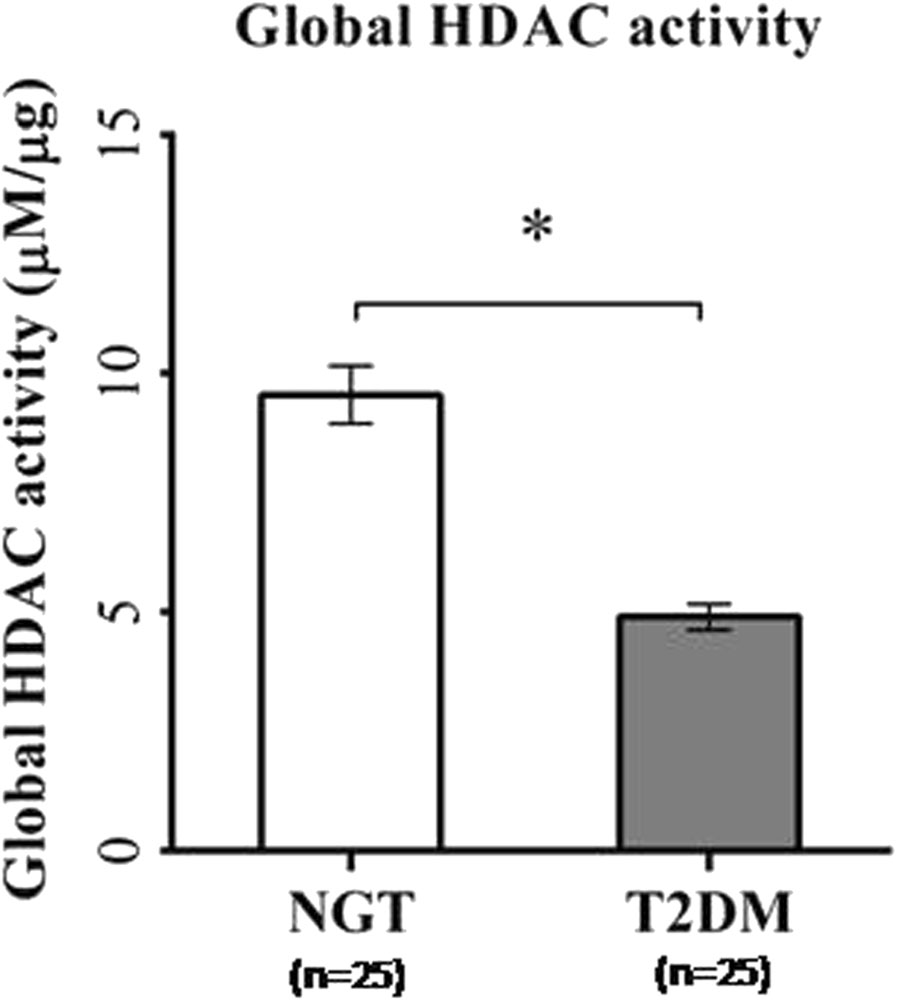
Global HDAC activity in PBMCs from control (NGT) subjects and type 2 diabetes mellitus (T2DM) patients. *Bars* represent the mean ± SEM, *p < 0.001 compared to control subjects

**Fig. 2:**
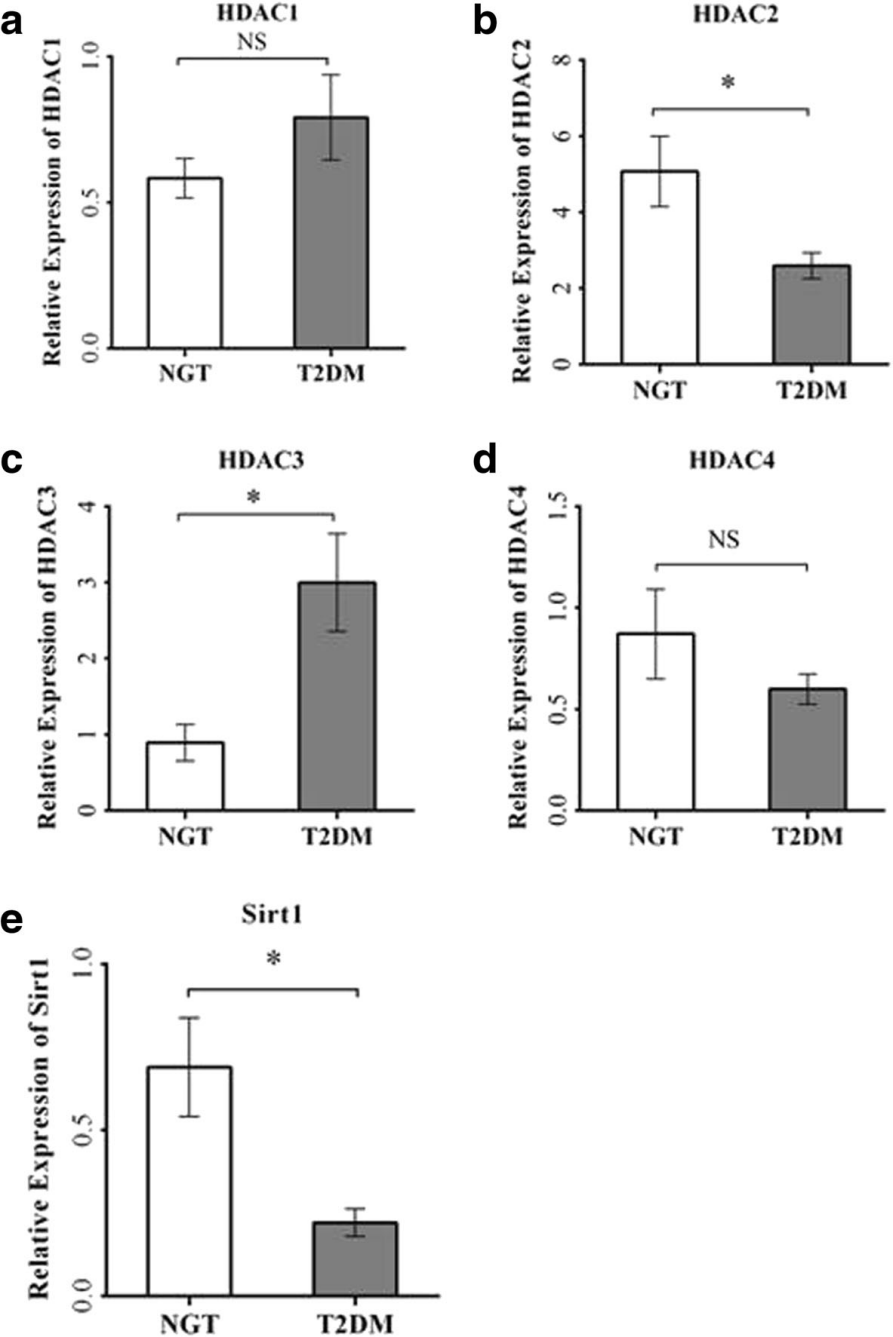
Real-time quantitative RT-PCR analysis of mRNA expression levels, viz., HDAC1 (**a**), HDAC2 (**b**), HDAC3 (**c**), HDAC4 (**d**), and Sirt1 (**e**) in PBMCs from control subjects and type 2 diabetes patients (*n* = 14 each). *Bars* represent the mean ± SEM, *p value <0.05 compared to control subjects, *NS* not significant

**Fig. 3:**
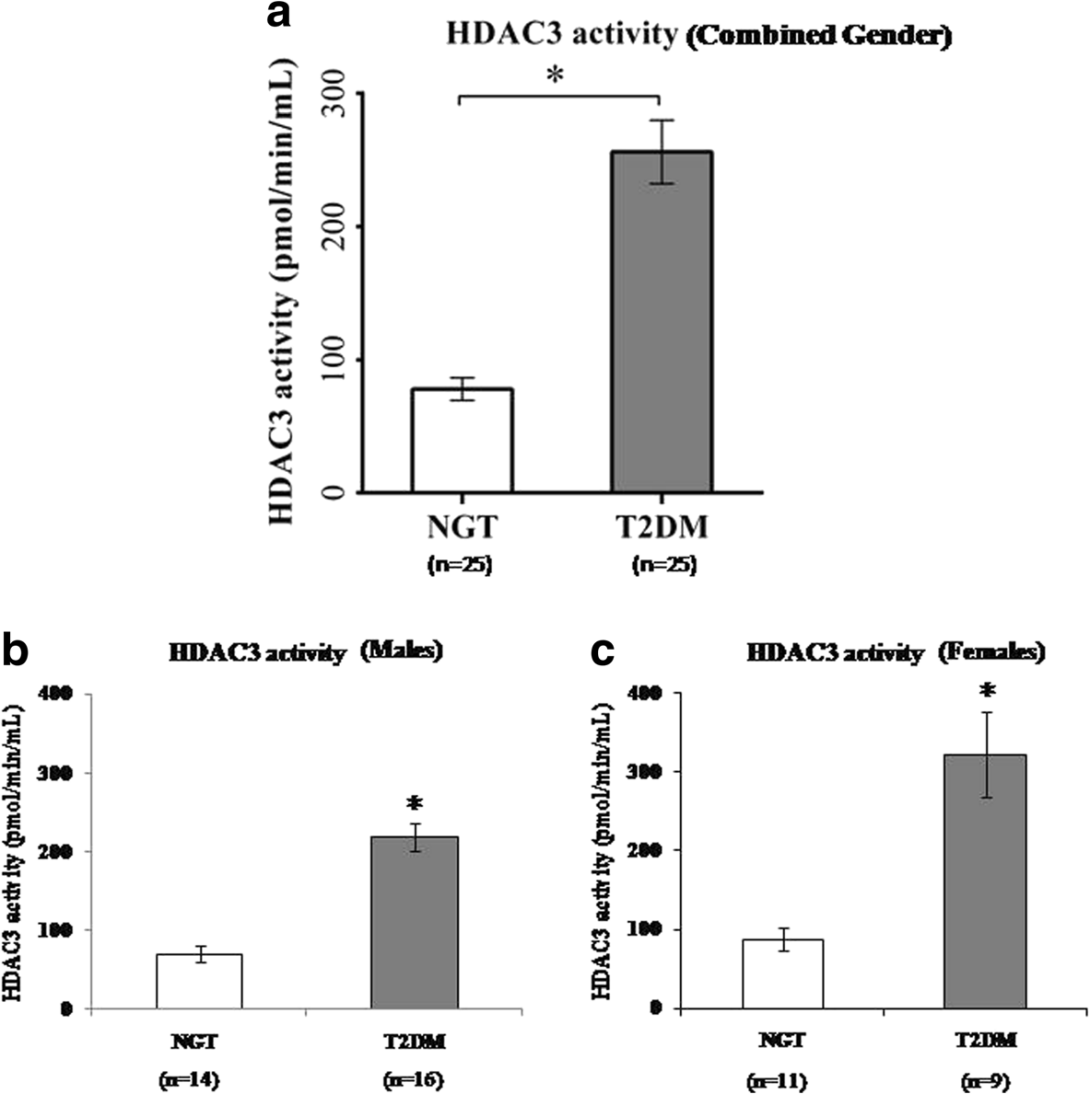
HDAC3 activity in PBMCs from control (NGT) subjects and type 2 diabetes mellitus (T2DM) patients—combined gender (**a**), males (**b**), and females (**c**). HDAC3 activity expressed in pmol/min/mL. *Bars* represent the mean ± SEM, *p < 0.001 compared to control subjects

The global histone deacetylase (HDAC) activity was significantly impaired (*p* < 0.001) in patients with type 2 diabetes compared to control subjects (Fig. 1). Then, we have also analyzed the individual subtypes of HDAC mRNA expression patterns (Fig. 2) from PBMCs in patients with type 2 diabetes compared to control subjects. Among the different subtypes of HDACs, *HDAC3* mRNA expression was significantly (*p* < 0.05) elevated and HDAC2 mRNA seen decreased (*p* < 0.05) in T2DM. Both HDAC1 and HDAC4 also showed altered levels (albeit not statistically significant) in T2DM at transcriptional levels. Sirt1 which represents the class III HDACs was decreased significantly (*p* < 0.05) in T2DM. As the highly significant HDAC3 mRNA expression was the prominent finding in our study in PBMCs from patients with type 2 diabetes, we then analyzed the specific HDAC3 activity in the study samples. Most strikingly, consistent with HDAC3 mRNA expression, PBMCs from patients with T2DM showed highly significant (*p* < 0.001) elevated levels of HDAC3 activity (Fig. 3a). Among the patients with type 2 diabetes, only two were on insulin in addition to OHAs. Analysis of data on HDAC3 activity, HDAC3 mRNA, and global HDAC activity showed that there were no differences in these parameters in T2DM with OHA alone compared to T2DM with OHA + insulin. When analyzed for gender, HDAC3 activity was increased (*p* < 0.001) to the extent of 2.7-fold in females (Fig. 3c) while it was 2.1-fold increased (*p* < 0.001) in male patients with T2DM (Fig. 3b) compared to their counterpart control subjects.

### Systemic and cellular levels of proinflammatory markers

**Fig. 4:**
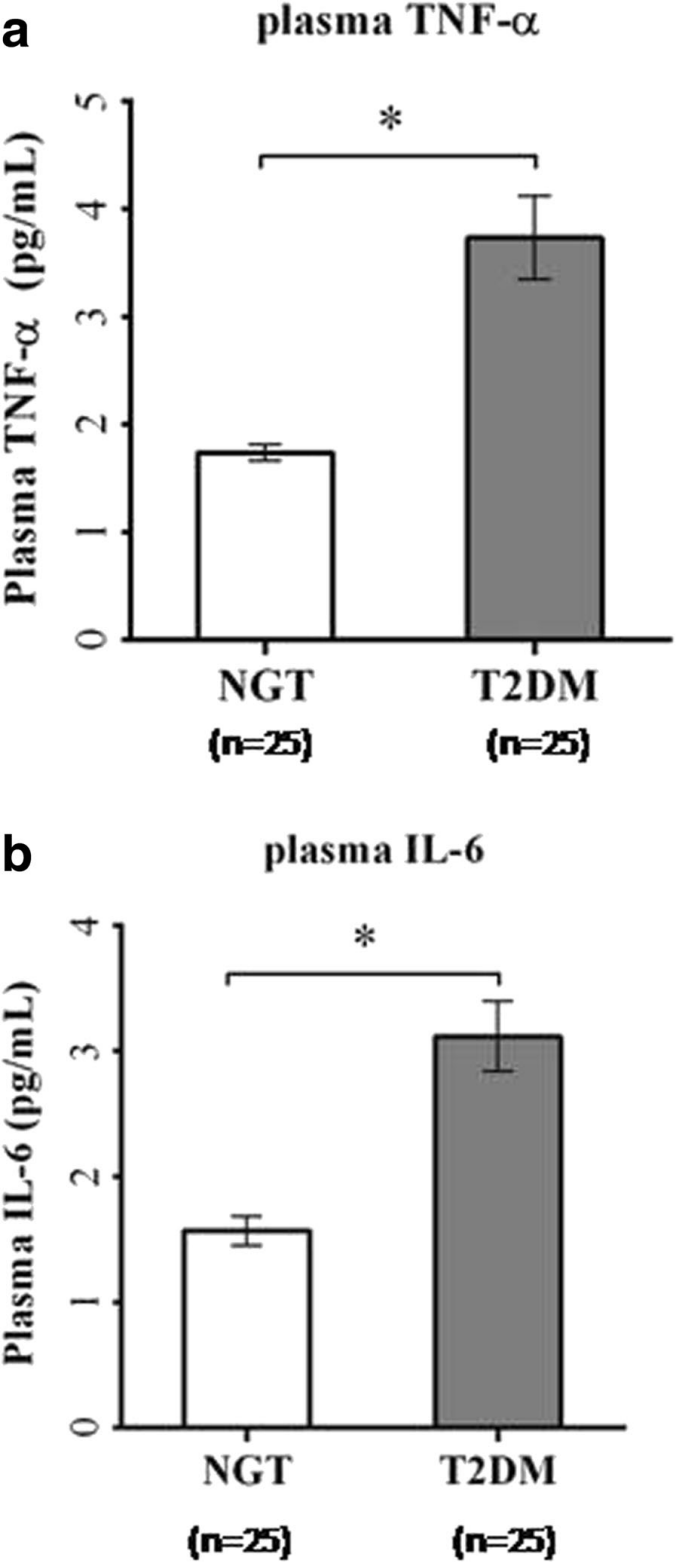
ELISA of plasma levels of TNF-α (**a**) and IL-6 (**b**) from control subjects and patients with type 2 diabetes. *Bars* represent the mean ± SEM, *p < 0.001 compared to control subjects

**Fig. 5:**
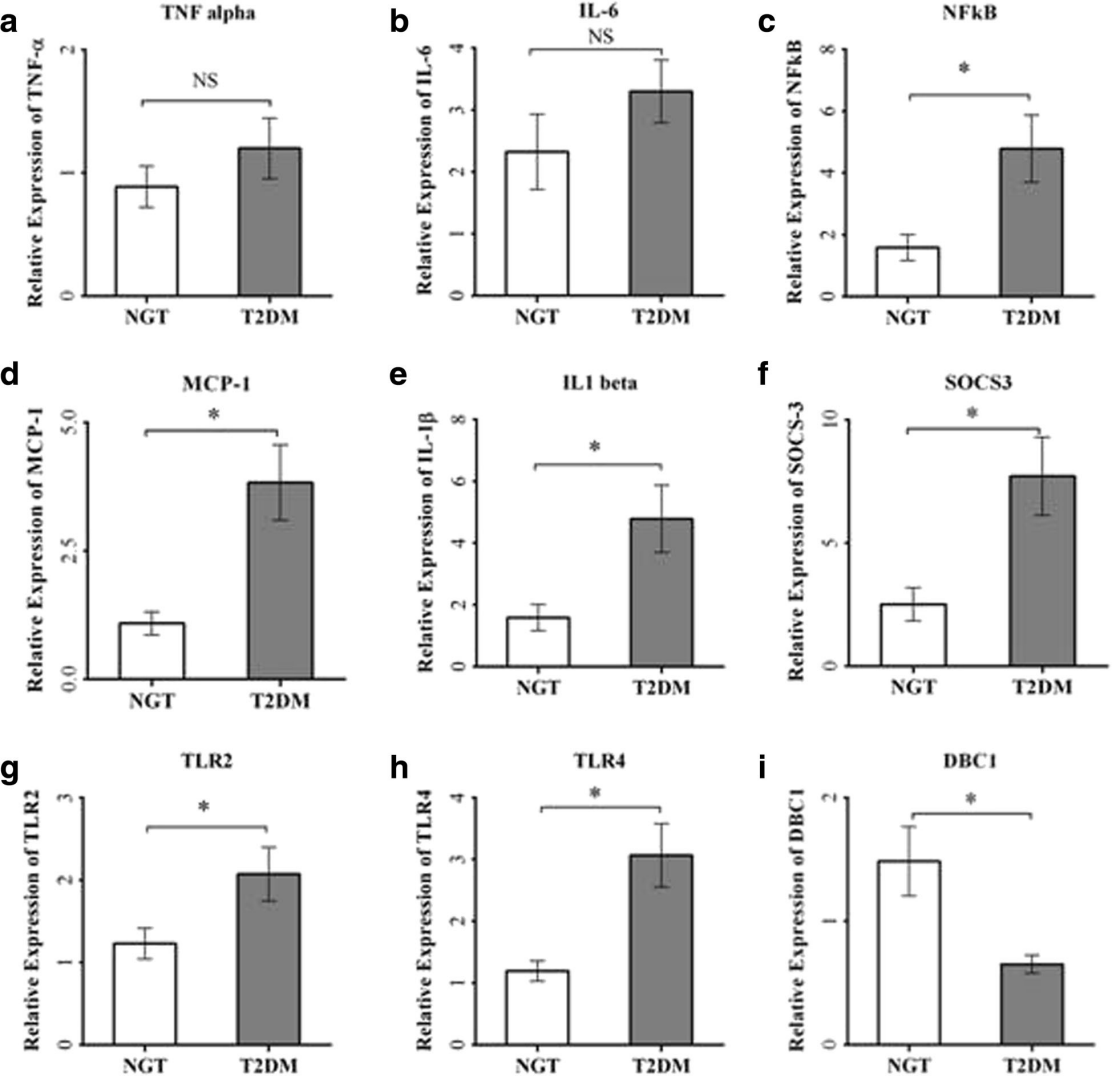
Quantitative real-time PCR analysis of mRNA expression levels, viz., TNF-α (**a**), IL-6 (**b**), NFκB (**c**), MCP-1 (**d**), IL1-β (**e**), SOCS-3 (**f**), TLR2 (**g**), TLR4 (**h**), and DBC1 (**i**) in PBMCs from control subjects and type 2 diabetes patients (*n* = 14 each). *Bars* represent the mean ± SEM; *p value <0.05 compared to control subjects; *NS* not significant

Plasma levels of both TNF-α (*p* < 0.001) and IL-6 (*p* = 0.002) were significantly higher in patients with type 2 diabetes compared to control subjects (Fig. 4a, b). Among the proinflammatory mediators, the mRNA expression of monocyte chemoattractant protein 1 (MCP-1), IL1-β, nuclear factor kappa-B (NFκB), Toll-like receptor 2 (TLR2), and Toll-like receptor 4 (TLR4) were significantly (*p* < 0.05) increased in T2DM (Fig. 5) while both the expression levels of TNF-α and IL-6 showed an increasing trend but not statistically significant. Interestingly, SOCS3 mRNA expression was significantly elevated (*p* < 0.05), and DBC1 mRNA expression was significantly (*p* < 0.05) decreased in PBMCs from T2DM patients (Fig. 5).

### HDAC3 activity/HDAC3 mRNA correlated with clinical, metabolic, and molecular risk factors

Pearson correlation analysis was done to check the relationship among *HDAC3* activity/HDAC3 mRNA and other risk variables (Table 2). *HDAC3* activity was significantly and positively correlated with fasting plasma glucose (*r* = 0.611, *p* < 0.001), HbA1c (*r* = 0.474, *p* = 0.001), and insulin resistance HOMA-IR (*r* = 0.526, *p* < 0.001) *HDAC3* activity also showed positive correlation with circulating TNF-α (*r* = 0.534, *p* < 0.001) and IL-6 (*r* = 0.617, *p* < 0.001). These associations are similar in both males and females. HDAC3 mRNA levels were also more or less similarly correlated to the above clinical and biochemical parameters. Pearson correlation analysis also revealed a strong positive correlation between the *HDAC3* activity and mRNA level of HDAC3 gene expression, various proinflammatory markers (MCP-1, NFκB, IL-1β, SOCS3, TLR2, TLR4), and negative association with Sirt1 and deleted in breast cancer 1 (DBC-1) transcriptional levels (Table 2). However, HDAC3 mRNA levels showed positive correlation only with transcriptional levels of MCP-1 and SOCS3. Logistic regression analysis using type 2 diabetes as dependent variable showed *HDAC3* activity or HDAC3 mRNA was associated significantly (*p* = 0.005) with T2DM, and this statistical significance was persisted even after adjusted for confounding factors like age and BMI. Interestingly, this association was lost when adjusted for insulin resistance, implying that the association between *HDAC3* and T2DM could be closely linked to insulin resistance state.

**Table 2:** Pearson correlation analysis of HDAC3 activity/HDAC3 mRNA expression with biochemical and molecular parameters in the study groups

Variables	HDAC3 activity		HDAC3 mRNA	
	*r* value	*p* value	*r* value	*p* value
Age (years)	−0.144	0.318	0.066	0.739
BMI (kg/m^2^)	0.208	0.147	0.218	0.265
Waist (cm)	0.028	0.865	0.121	0.602
Systolic blood pressure (mmHg)	0.098	0.498	0.233	0.233
Diastolic blood pressure (mmHg)	−0.015	0.916	0.199	0.31
Fasting plasma glucose (mg/dL)	0.611	<*0.001*	0.602	*0.001*
HbA1c (%)	0.474	*0.001*	0.447	*0.017*
HOMA-IR	0.526	<*0.001*	0.378	*0.047*
Plasma TNF-α	0.534	<*0.001*	0.417	*0.027*
Plasma IL-6	0.617	<*0.001*	0.381	*0.045*
Serum cholesterol (mg/dl)	−0.181	0.210	0.044	0.825
Serum triglycerides (mg/dl)	0.106	0.465	0.152	0.439
LDL cholesterol (mg/dl)	−0.19	0.185	0.023	0.908
HDL cholesterol (mg/dl)	−0.161	0.263	−0.052	0.792
TNF-α mRNA expression	0.175	0.374	0.027	0.892
IL-6 mRNA expression	0.167	0.395	−0.016	0.935
NFκB mRNA expression	0.627	<*0.001*	0.118	0.559
IL1-β mRNA expression	0.643	*0.001*	0.247	0.255
MCP-1 mRNA expression	0.521	*0.004*	0.846	<*0.001*
SOCS3 mRNA expression	0.708	<*0.001*	0.557	*0.002*
TLR2 mRNA expression	0.484	*0.009*	0.156	0.437
TLR4 mRNA expression	0.444	*0.018*	0.234	0.240
DBC1 mRNA expression	−0.396	*0.037*	−0.315	0.109
HDAC3 mRNA expression	0.549	*0.002*	–	–
HDAC3 activity	–	**–**	0.549	*0.002*
Sirt1 mRNA expression	−0.414	*0.029*	−0.125	0.527

Italic values are to indicate the statistical significance

## Discussion

While there is a great demand in looking for new drug targets and development of novel therapeutic measures (with different mode of actions) for type 2 diabetes, the present study assumes significance for the following reasons. Firstly, this clinically relevant study demonstrated an association of elevated *HDAC3* activity and HDAC3 mRNA expression in PBMCs from patients with T2DM. Secondly, increased *HDAC3* activity/HDAC3 mRNA level was positively correlated to all the inflammatory markers profiled, poor glycemic control, and insulin resistance. Thirdly, HDAC3 activity exhibited a negative association with transcriptional levels of Sirt1, suggesting an imbalance of HDAC3/Sirt1 circuit. Finally, the transcriptional level of DCB1 (which supposed to negatively regulate HDAC3) was significantly decreased in patients with T2DM.

Tracing molecular events through different layers of biological information, including histone modifications and physiological data, is required for solving the puzzle of the etiology of type 2 diabetes and other metabolic disorders. Several animal and cell model studies have emphasized that class I HDAC inhibition could promote the mitochondrial biogenesis, induce oxidative metabolism, and beneficially alter glucose and lipid metabolism [13, 17]. Studies by Larsen et al. [18] and Lundh et al. [19] have revealed that HDAC inhibition prevents cytokine-induced β cell apoptosis and impaired β cell function associated with a downregulation of NFκB transactivating activity. Genetic silencing or pharmacological inhibition of HDAC isoforms 1, 2, and 3 have been shown to increase MKP-1 acetylation and decrease LPS-induced MAPK-p38 phosphorylation and TNF-α, IL1-β, NOS2, and nitrate synthesis [20]. In our study, we observed increased HDAC1 mRNA expression (albeit not statistically significant) in patients with type 2 diabetes. In patients with type 2 diabetes, we also observed significantly decreased HDAC2 mRNA expression in PBMCs. Earlier, it has been shown that HDAC2 expression was reduced in endothelial cells when exposed to oxLDL, and HDAC2 also decreased in the atherosclerotic lesions of human coronary arteries [21]. While the functional significance of alterations in HDAC1 and HDAC2 needs to be clarified in metabolic disorders, our study unraveled a consistent increase in both HDAC3 activity and HDAC3 transcriptional levels in PBMCs from patients with type 2 diabetes. As PBMCs serve as a potentially accessible surrogate cell model to study the pathogenesis of diabetes and its complications [22], our study using PBMCs as a proxy for demonstrating the epigenetic alterations with special reference to HDAC3 and inflammation is significantly important as these cells infiltrate peripheral organs/tissues and culminate in the etiology of type 2 diabetes.

In our study, *HDAC3* activity is increased several folds in the PBMCs from patients with type 2 diabetes compared to control subjects and it showed strong positive correlation with systemic TNF-α and IL-6; mRNA expressions of MCP-1, SCOS3, and *HDAC3*; and fasting glucose, HbA1c, and insulin resistance (HOMA-IR), while exhibited a negative association with Sirt1. The correlation of HDAC3 mRNA levels with various clinical and molecular parameters observed in our study also implies that this could have important implications for HDAC3’s role as a co-factor in many different protein complexes beyond its deacetylase activity. Among the proinflammatory markers, the mRNA expression of MCP-1, NFκB, TLR2, TLR4, and IL1-β were significantly increased in T2DM while both the expression levels of TNF-α and IL-6 showed an increasing trend. Interestingly, SOCS3 mRNA expression was significantly elevated in T2DM patients, and this feedback regulation might have mirrored the extent of mRNA expression of both TNF-α and IL-6 seen in our study. In fact, accumulating literature favors inhibition of HDAC3 as a potential strategy for developing novel diabetes therapeutics [17, 23]. Using an integrated genomic approach, Chen et al. [24] found that *HDAC3* deficient macrophages were not able to activate almost half of the inflammatory gene expression program when they are stimulated with LPS and concluded that *HDAC3* is required for the activation of innate immunity. High expression of HDAC3 in the liver has been shown to contribute to high-fat diet-induced metabolic syndrome by suppressing the PPAR-γ and LXR-α-pathways in rats [25]. Interestingly, conditional deletion of HDAC3 in osteoprogenitor cells has been shown to attenuate high-fat diet-induced insulin resistance, hepatic steatosis, and metabolic syndrome [26]. While HDAC3 was the sole HDAC isoform upregulated in ruptured lesions of atherosclerosis, HDAC3 deletion has been shown to shift plaque macrophages to an anti-inflammatory phenotype and reduce lipid accumulation [27]. Interestingly, three single nucleotide variants of the HDAC3 gene were shown associated with type 2 diabetes mellitus in a Chinese population [28]. Very recently using a selective HDAC3 inhibitor (BRD3308), Lundh et al. [29] have demonstrated reduction in hyperglycemia and increase in insulin secretion in a rat model of type 2 diabetes. It has been expected that HDAC inhibitors can elicit transgenerational effects, via cross-talk between different epigenetic mechanisms, to have an impact on disease phenotypes in a beneficial manner. Indeed, Jia et al. [30] have demonstrated that selective HDAC inhibition (targeting HDAC1 and 3) imparted beneficial transgenerational effects in Huntington’s disease mice via altered DNA and histone methylation.

Consistent with the protective role of Sirt1 (class III HDAC) demonstrated in the literature [31–33], Sirt1 mRNA levels were significantly decreased in PBMCs from patients with type 2 diabetes. Sirt1 protects β cells from cytokine-mediated cytotoxicity by altering NO production and iNOS expression by inhibition of NFκB through P53 pathway [34]. Reduction of Sirt1 has been shown to cause macrophage recruitment and infiltration into the adipose tissue [35–37]. Since Sirt1 is a class III HDAC and it plays a beneficial role in glucose/lipid homeostasis and energy expenditure, non-specific inhibition of all HDACs would have drastic consequences. Another interesting observation in our study is that mRNA levels of DBC1 (deleted in breast cancer 1) were significantly reduced in PBMCs from patients with type 2 diabetes compared to control subjects. DBC1 serves as an endogenous inhibitor HDAC3 [38], and it plays a crucial physiological role as a modulator of epigenetics and metabolic function. In an earlier study, DBC1 knockout mice showed increased expression of PEPCK and gluconeogenesis, whereas over expression DBC1 reduced the PEPCK expression [39]. As we saw a negative correlation between HDAC3 activity and mRNA levels of DBC1 in our study, it appears that the regulatory circuit of endogenous control of HDAC3 could be somehow lost in patients with type 2 diabetes and this needs to be studied in-depth in future investigations.

It is possible that HDACs could negatively regulate certain miRNAs and thereby mediate proinflammation. Recently, increased HDAC3 and TNFα and decreased miRNA-130a expression has been observed in PBMCs from patients with spinal cord injuries [40]. We observed an impairment of miR-146a linked with insulin resistance and inflammation in patients with type 2 diabetes [7]. Interestingly, HDAC inhibitors have been shown to increase miR-146a expression and negatively regulate interleukin-1β signaling in osteoarthritis [41]. These studies emphasize HDAC3/miRNA axis as a novel regulator of inflammation in type 2 diabetes which should be exploited for novel therapeutic measures.

Having seen the HDAC alterations in the clinical setting of diabetes, it would be interesting to probe and understand whether HDAC regulation by endogenous mediators is also altered in type 2 diabetes. Butyrate is endogenously made in the human body by anaerobic bacterial fermentation of carbohydrates (derived from dietary fiber) in the colon and it has been shown as an inhibitor of HDACs with anti-inflammatory activity [42–44]. High-fat diet has been shown to reduce the formation of butyrate [45], and administration of butyrate to diabetic rats reduced insulin resistance and improved glucose tolerance [46, 47]. Chang et al. [48] have also shown that the microbial metabolite butyrate regulates intestinal macrophage function via histone deacetylase inhibition. More importantly from several studies, it has been now realized that butyrate-producing bacterial strain concentrations were lower in patients with type 2 diabetes [49–51]. Therefore, it appears that dietary interventions influencing microbial composition and thereby beneficially altering endogenous butyrate levels and/or free fatty acid receptors (FFARs) may be considered as an option in the prevention and treatment of type 2 diabetes and other metabolic disorders. While the benefits of physical exercise on type 2 diabetes and prevention and control is well conceived, recent studies imply that such beneficial metabolic changes can occur through epigenetic alterations like histone modifications [52]. Exercise benefits at the molecular level are linked, in part, to transcriptional activation myocyte-specific enhancer factor (MEF) 2A in coordination with PPAR-γ, PPARGC1a, and acetylation of GLUT4 in the skeletal muscle [53]. It has been recently shown that exercise increases the binding of MEF2A to the Cpt1b promoter in the mouse skeletal muscle and this requires repression of MEF2A-binding partners such as HDAC5 and HDAC3 [54]. Interestingly, a short-term intensive practice of mindfulness meditation has been demonstrated to rapidly decrease histone deacetylases (more strikingly HDAC3) and inflammatory gene expression [55] implying that appropriate lifestyle modifications may offer metabolic benefits operating through epigenetic mechanisms.

## Conclusions

To conclude, in a relevant clinical diabetes setting, we have demonstrated increased *HDAC3* activity/HDAC3 mRNA levels in patients with type 2 diabetes that was positively correlated to all the inflammatory markers profiled, poor glycemic control, and insulin resistance. The striking message from our study is that while looking for antidiabetic drugs with newer mode of action as well as additional anti-inflammatory benefits, the development of selective isoform-specific *HDAC3* inhibitors (with appropriate pharmacokinetics and dynamics) appear to have potential advantage.

## Abbreviations

BMI: Body mass index
DBC-1: Deleted in breast cancer 1
HDAC: Histone deacetylase
HDAC3: Histone deacetylase 3
HOMA-IR: Homeostatic model assessment
IL-1β: Interleukin-1β
IL-6: Interleukin-6
MCP-1: Monocyte chemoattractant protein 1
NFkB: Nuclear factor kappa-B
NGT: Normal glucose tolerance
PBMC: Peripheral blood mononuclear cell
Sirt1: Sirtuin1
SOCS-3: Suppressor of cytokine signaling 3
T2DM: Type 2 diabetes mellitus
TLR2: Toll-like receptor 2
TLR4: Toll-like receptor 4
TNF-α: Tumor necrosis factor-α
